# Dietary Fibre Improves First-phase Insulin Secretion in Overweight Individuals

**DOI:** 10.1371/journal.pone.0040834

**Published:** 2012-07-16

**Authors:** Caroline L. Bodinham, Leanne Smith, John Wright, Gary S. Frost, M. Denise Robertson

**Affiliations:** 1 Department of Nutrition and Metabolism, University of Surrey, Guildford, United Kingdom; 2 Nutrition & Dietetic Research Group, Imperial College London, London, United Kingdom; Paris Institute of Technology for Life, Food and Environmental Sciences, France

## Abstract

Previous work has shown increased insulin sensitivity, increased hepatic insulin clearance and lower postprandial insulin responses following treatment with resistant starch, a type of dietary fibre. The objective of this study was to further explore the effects of resistant starch on insulin secretion. Twelve overweight (BMI 28.2±0.4 kg/m^2^) individuals participated in this randomized, subject-blind crossover study. Participants consumed either 40 g type 2 resistant starch or the energy and carbohydrate-matched placebo daily for four weeks. Assessment of the effect on insulin secretion was made at the end of each intervention using an insulin-modified frequently sampled intravenous glucose tolerance test (FSIVGTT). Insulin and C-peptide concentrations were significantly higher during the FSIVGTT following the resistant starch compared with the placebo. Modelling of the data showed significantly improved first-phase insulin secretion with resistant starch. These effects were observed without any changes to either body weight or habitual food intake. This study showed that just four weeks of resistant starch intake significantly increased the first-phase insulin secretion in individuals at risk of developing type 2 diabetes. Further studies exploring this effect in individuals with type 2 diabetes are required.

## Introduction

Over recent years the incidence of type 2 diabetes has increased and with this rise there is an associated increase in treatment strategies to help combat the problem. Those that help to prevent the development of impaired glucose tolerance (often referred to as pre-diabetes) and the progression from pre-diabetes to diabetes are clearly important.

Currently, the recommendation for treatment of pre-diabetes is with diet and lifestyle modifications and annual screening for progression to diabetes (ADA Guidelines, 2011) [1]. In some cases where there are co-morbidities or where an individual is at high risk of developing diabetes (such as individuals with a strong family history or who are obese), individuals may be treated with metformin (ADA Guidelines, 2011) [1]. Clearly, any dietary intervention that can help to improve insulin secretion or sensitivity and glucose control would be of value.

Loss of first-phase insulin secretion has been well characterised as a primary defect in the development of type 2 diabetes [2], [3]. Restoration and improvement of first-phase insulin secretion is an important target in the treatment of type 2 diabetes and is a major property of sulphonylureas which have been shown in some studies to almost double the first-phase insulin response [4], [5].

Several studies have shown that consumption of a non-viscous dietary fibre in the form of resistant starch (RS) may affect glucose and insulin concentrations when matched with controls of identical glycaemia [6]–[9]. A previous study by our group in individuals with insulin resistance (pre-diabetes) demonstrated that consumption of 40 g/day type 2 RS derived from high amylose maize (HAM-RS2) for 12 weeks increased tissue insulin sensitivity by 19%, compared with an energy and carbohydrate-matched placebo [9]. Our group has shown a reduced postprandial insulin response following acute (24 hours) ingestion of HAM-RS2 [6]; whilst the study showed an increased C-peptide:insulin ratio, implying improved hepatic insulin clearance, the study design did not provide information as to whether consumption of RS might affect insulin secretion. The current study was therefore designed to further explore the effect of HAM-RS2 utilising an insulin-modified frequently sampled intravenous glucose tolerance test (FSIVGTT) to determine whether the effects of RS are due to improvements in insulin secretion or clearance.

## Methods

A total of 12 overweight individuals (8 males and 4 females; mean age 37±4.0 years, BMI 28.2±0.4 kg/m^2^) with insulin resistance (fasting insulin 96±9.7 pmol/l) as defined by the European Group for the Study of Insulin Resistance (EGIR) criteria [10], but without a diagnosis of T2DM were recruited. These diagnostic criteria were chosen as the most discriminating for the presence of insulin resistance [11]. The participants had no history of gastrointestinal disease and were not following any dietary restrictions. The study commenced in 2008 and was conducted according to the guidelines laid down in the Declaration of Helsinki, and all procedures were approved by the Surrey NHS Research Ethics Committee (REC reference 08/H1109/112) and the University of Surrey Research Ethics Committee (EC/2008/80/FHMS). Written informed consent was obtained from all participants.

The study was a subject-blind, randomised crossover study. Participants consumed either 67 g Hi-maize 260® (60% resistant starch and 40% rapidly digestible starch (RDS) providing 40 g HAM-RS2 as measured by The Association of Official Analytical Chemists for total dietary fibre method 991·43) or 27 g Amioca® (100% RDS) daily for 4 weeks, separated by a 4 week washout period. Both supplements were supplied by the National Starch Company, LLC (Bridgewater, NJ, USA) in ready-to-use sachets that simply required mixing into a cold liquid.

During the final week of each intervention, participants completed 7-day dietary records to assess food intake and a 7-day bowel habit and symptom diary to assess gastrointestinal tolerance of the supplements.

At the end of each intervention, participants attended the CEDAR Centre at the Royal Surrey County Hospital for an insulin-modified frequently sampled intravenous glucose tolerance test (FSIVGTT). Participants arrived fasting and following voiding, body weight and composition were measured by bioimpedance (Tanita, Arlington Heights, IL, USA). Blood pressure was taken after resting for 5 minutes, and the mean of 3 readings was recorded (Omron MX3 Plus, Omron Healthcare Europe, Milton Keynes, United Kingdom).

An indwelling intravenous cannula was inserted into each arm, one for sampling and the other for infusion. At time zero, a glucose bolus was administered at a dose of 0.3 g/kg body weight (maximum dose of 25 g glucose) over 5 minutes, followed, at time 20 minutes, by a bolus of insulin (Actrapid, Novo Nordisk Denmark) at a dose of 0.03 U/kg body weight given over 5 minutes. Frequent blood samples were taken (a total of 29 samples) for a total of 3 hours.

Blood glucose concentrations were measured immediately using the YSI 2300 STAT Plus™ (YSI Life Sciences, UK). Plasma insulin and C-peptide concentrations were measured by radioimmunoassay using commercially available kits (Millipore, Billerica, MA), inter-assay CV <10%. Fasting plasma lipid concentrations were measured using commercially available kits, triacylglycerides (TAG) and total cholesterol using IL Test kits (Insturmentation laboratory, UK); and non-esterified fatty acids (NEFA) using RANDOX kits (RANDOX Laboratories Ltd, UK), for the ILab650 (Instrumentation Laboratory, UK).

All statistical analyses were carried out using SPSS 16.0 for Windows (Chicago, USA). Statistical significance was taken as *p*<0.05. The raw glucose, insulin and C-peptide data were analysed using repeated measures ANOVA. The data were also modelled using Bergman’s minimal model (MINMOD Millennium version), which has been described by Boston *et al* [12]. All results are expressed as mean ± SEM.

## Results

Both HAM-RS2 and placebo were well tolerated as reported in the bowel habit and symptom diaries. There were no significant differences between the HAM-RS2 and placebo for body weight, adiposity, waist circumference or blood pressure (Table 1). Fasting glucose concentrations were significantly lower following 4 weeks supplementation with HAM-RS2 compared with placebo (*p = *0.049), but there were no significant differences between the supplements for fasting insulin, C-peptide or lipid concentrations (Table 1).

**Table 1:** Anthropometric measurements and fasting plasma concentrations taken after 4 weeks daily supplementation with either 40 g/day of HAM-RS2 or placebo.

	HAM-RS2	Placebo	*P value*
Weight (kg)	86.8 (2.1)	87.1 (2.2)	*NS*
BMI (kg/m^2^)	28.4 (0.5)	28.4 (0.5)	*NS*
Body Fat (%)^1^	28.1 (2.2)	27.8 (2.2)	*NS*
Waist Circumference (cm)	98.3 (1.2)	98.7 (1.0)	*NS*
Systolic Blood Pressure (mmHg)^2^	122 (3)	120 (3)	*NS*
Diastolic Blood Pressure (mmHg)^2^	77 (2)	74 (3)	*NS*
Fasting Glucose (mmol/l)	4.8 (0.1)	5.0 (0.1)	*0.049*
Fasting Insulin (pmol/l)	88.6 (9.5)	85.4 (7.8)	*NS*
Fasting C-peptide (nmol/l)	0.84 (0.1)	0.86 (0.1)	*NS*
Fasting TAG (mmol/l)	1.6 (0.3)	1.3 (0.2)	*NS*
Fasting NEFA (mmol/l)	0.59 (0.1)	0.48 (0.1)	*NS*
Total Cholesterol (mmol/l)	4.8 (0.3)	4.8 (0.3)	*NS*

All values are mean (SEM), N = 12. Comparisons made with paired t-tests.

1 Measured by bioimpedance (Tanita, Arlington Heights, IL, USA). N = 11.

2 Mean of 3 readings taken with the subject in a sitting position, measured by an automatic blood pressure cuff (Omron MX3 Plus, Omron Healthcare Europe, Milton Keynes, United Kingdom).

Analysis of the 7-day dietary records revealed no significant differences between the HAM-RS2 and placebo for either energy or macronutrient intakes. Fibre intake was significantly higher during the HAM-RS2 intervention compared with the placebo (57.8±1.2 g/day versus 17.5±1.6 g/day, respectively; *p* = <0.001) which can be directly attributed to the HAM-RS2 supplement.

**Figure 1:**
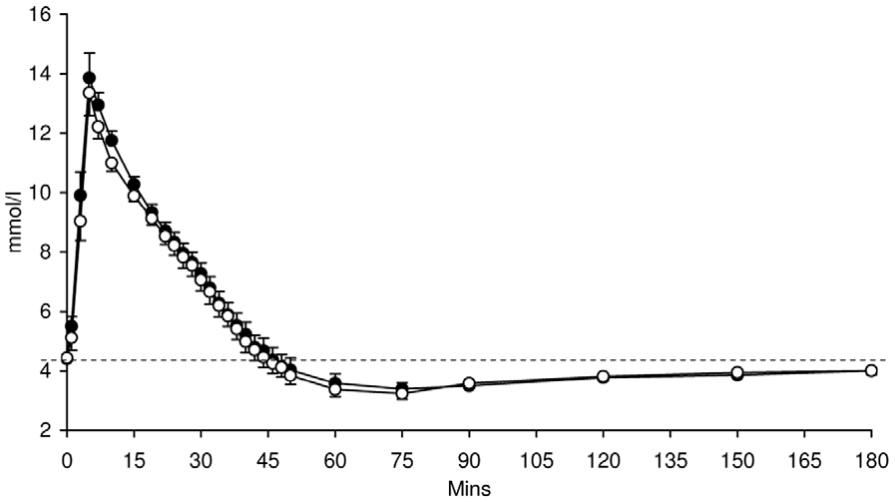
Glucose concentrations from FSIVGTT after 4 weeks daily supplementation with 40 g HAM-RS2 or placebo. N = 12, mean ± SEM. No significant difference between the HAM-RS2 and placebo. Comparisons made with repeated measures ANOVA. Black circles  =  HAM-RS2; white circles  =  placebo; dashed line  =  baseline glucose concentrations.

**Figure 2:**
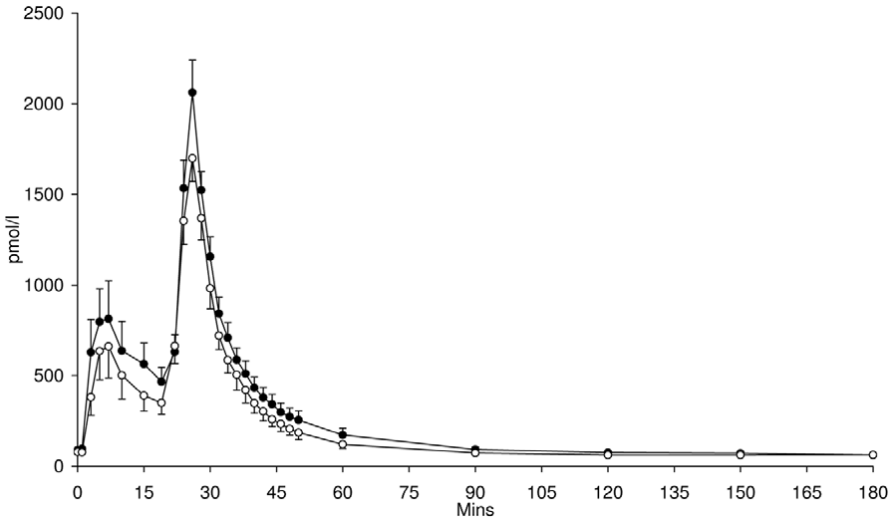
Insulin concentrations from FSIVGTT after 4 weeks daily supplementation with 40 g HAM-RS2 or placebo. N = 12, mean ± SEM. Significantly higher concentrations following HAM-RS2 compared with placebo (*p* = 0.009). Comparisons made with repeated measures ANOVA. Black circles  =  HAM-RS2; white circles  =  placebo.

**Figure 3:**
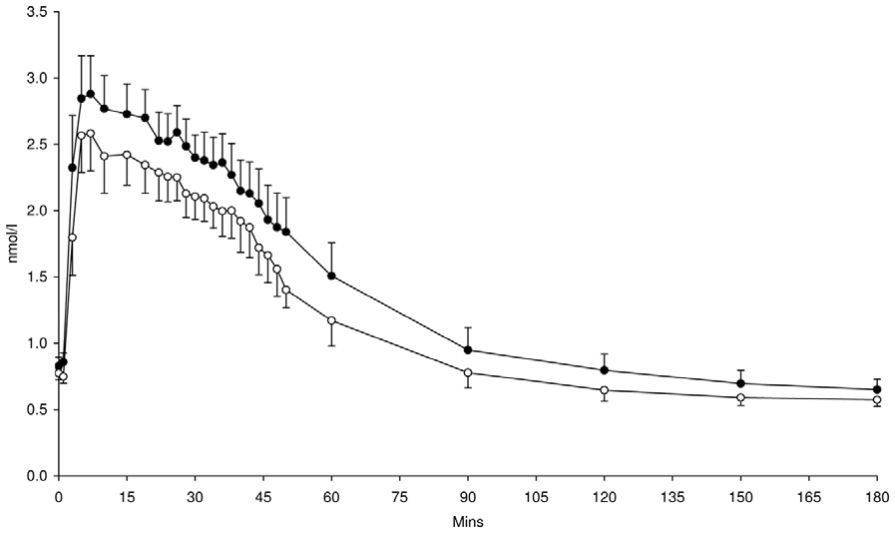
C-peptide concentrations from FSIVGTT after 4 weeks daily supplementation with 40 g HAM-RS2 or placebo. N = 12, mean ± SEM. Significantly higher concentrations following HAM-RS2 compared with placebo (*p* = 0.016). Comparisons made with repeated measures ANOVA. Black circles  =  HAM-RS2; white circles  =  placebo.

Blood glucose concentrations during the FSIVGTT were not significantly different following supplementation with either HAM-RS2 or placebo (Figure 1). However, plasma insulin (Figure 2) and C-peptide (Figure 3) concentrations were significantly higher following 4 weeks supplementation with HAM-RS2 compared with placebo (*p* = 0.009 and *p* = 0.016, respectively).

Whilst modelling of the data showed no significant difference between the supplements for insulin sensitivity or disposition index, there was a significantly higher first-phase insulin response (AIRg) following supplementation with HAM-RS2 compared with placebo and a trend for increased glucose effectiveness with the HAM-RS2 (Table 2).

**Table 2:** Indices from the modelling of the data from the IVGTT.

	HAM-RS2	Placebo	*P value*
Insulin Sensitivity ((mu/l)−1 min−1)	3.3 (0.6)	3.6 (0.5)	*NS*
First Phase Insulin (AIRg)(mu.l−1 min)	859.9 (244.5)	634.5 (191.4)	*0.009*
Disposition Index	2526.5 (732.2)	2235.3 (740.6)	*NS*
Glucose Effectiveness (min−1)	0.03 (0.003)	0.02 (0.003)	*0.06*

All values are mean (SEM), N = 12. Comparisons made with paired t-tests.

## Discussion

This study was designed to further explore the effects of HAM-RS2 on insulin secretion. To our knowledge this is the first study to demonstrate a significant improvement in first-phase insulin secretion following short-term supplementation with dietary fibre in the form of resistant starch (HAM-RS2). This work adds to our group’s previous findings of a positive effect of HAM-RS2 on insulin sensitivity [7], [8].

Loss of first-phase insulin secretion is a well-characterised defect in type 2 diabetes [2] and improvement in this following a simple dietary intervention has potentially important clinical implications. Sulphonylurea drugs have long been a mainstay in the treatment of type 2 diabetes. A study by Hosker *et al* [4] investigated the first and second phase beta cell responses in individuals without diabetes and those with type 2 diabetes controlled by diet alone. During hyperglycemic clamps at 3 glucose levels (7.5, 10 and 15 mmol/l), the first and second phase insulin responses were impaired in those with diabetes, and treatment with a sulphonylurea (gliclazide) in those with diabetes resulted in restoration of both the first and second phase insulin responses, with an approximate doubling of the insulin and C-peptide response at each level of clamp. In the current study consumption of HAM-RS2 for 4 weeks increased the first-phase insulin response by 36% compared with placebo treatment. The fact that this increase approaches that seen with sulphonylurea treatment suggests that HAM-RS2 may have potential as a strategy to prevent the progression from impaired glucose tolerance to type 2 diabetes and, indeed, in the treatment of type 2 diabetes.

To our knowledge dietary fibre has been shown to increase insulin secretion only once before. In a study by Juntunen *et al* [13] 8 weeks consumption of high fibre rye bread significantly increased acute insulin secretion by 7.1% compared to 8 weeks of white wheat bread in healthy postmenopausal women. It has been suggested that components specific to rye bread such as phenolic acids or tannins may be responsible; however, as HAM-RS2 contains neither of these compounds this seems unlikely as a mechanism.

Our study does not provide data to explain the mechanism(s) for the effect of HAM-RS2 on insulin secretion although this is likely to be through multi-system effects. As the improvement is similar to that seen with sulphonylureas, it is possible that RS may work by similar mechanisms. Sulphonylureas bind to beta cell membrane receptors which result in the increase in insulin secretion. Although there is no direct evidence for products of HAM-RS2 digestion binding to beta cell membrane receptors and activating signalling pathways in a similar manner to sulphonylureas, recent work in animal models with HAM-RS2 has shown an increased pancreatic beta cell density in experimental diabetes [14]. The increase in SCFA concentrations following fermentation of RS may also lead to other possible mechanisms including changes in ectopic TAG storage within the pancreas; however, changes in ectopic TAG storage may take 12 weeks of HAM-RS2 treatment to become evident [9]. Colonic SCFA production has been linked to increases in the secretion of incretin hormones (such as glucagon-like peptide-1, GLP-1) from enteroendocrine cells and therefore may be an alternative mechanism for an increase in first-phase insulin secretion. However, whilst there are data from rodent studies showing increases in GLP-1 following RS intake [15]–[17] data confirming this effect in humans are lacking, and indeed, one study in humans has shown that it may take a year of increased fibre intake (increase of 20 g/day) to increase GLP-1 secretion [18]. It is therefore unlikely that increases in incretin hormones alone are the mechanism behind the effect. Circulating SCFA, in particular propionate, may also increase insulin secretion through binding to PPARγ receptors in adipose tissue [19] and activating pathways similar to those of the thiazolidinediones [20]. Further mechanistic studies are clearly required to investigate these potential mechanisms.

Whereas studies in obese individuals with type 2 diabetes have shown that following bariatric surgery first-phase insulin can be restored [21], the improvement observed in our study was independent of any changes in body weight or lifestyle. Similarly there were no effects on plasma lipid concentrations. These results confirm the results of previous studies by our group in healthy individuals and those with the metabolic syndrome [7], [9], as well as in work by other groups using different types of RS.

Although this study showed an improvement in insulin secretion following HAM-RS2, it did not demonstrate an improvement in insulin sensitivity which we have shown previously using the hyperinsulinemic-euglycemic clamp [7]–[9]. Our group have only previously investigated the effects of HAM-RS2 in individuals with the metabolic syndrome once and this study was of 12 weeks duration [9]; it may therefore be that the current study of 4 weeks maybe too short a time frame for effects on insulin sensitivity to occur and that the improvement in first-phase insulin secretion may be an early step in this improvement of insulin sensitivity. The hyperinsulinemic-euglycemic clamp is the gold-standard direct measure of insulin sensitivity or insulin-mediated glucose disposal, whilst the IVGTT is less robust due to the hypoglycaemia and gluco-regulatory hormone release invoked by the insulin infusion (as clearly seen in Figure 1), especially in those without diabetes. However, the IVGTT may be ideally suited to the assessment of first-phase insulin secretion. We would anticipate that an improvement in first-phase insulin might be associated with an improvement in insulin sensitivity; however, the time-course of this relationship needs further investigation.

In conclusion, we have shown that a simple, inexpensive, well tolerated, non-pharmacological dietary supplementation with HAM-RS2 can improve first-phase insulin secretion in overweight individuals who are at increased risk of developing type 2 diabetes. Further studies are required to confirm these findings and to elucidate the mechanisms. It would also warrant further investigation in individuals with diabetes, as no results have been published in humans exploring the effects of HAM-RS2 treatment in type 2 diabetes.
